# Impact of structure and formulation changes on the function of insulin products

**DOI:** 10.3389/fendo.2025.1601119

**Published:** 2025-12-29

**Authors:** YeonJin Yang, Md. Shahinozzaman, Hyunsu Shin, Sujata Bupp, Carole Sourbier

**Affiliations:** Office of Pharmaceutical Quality Research, Office of Pharmaceutical Quality, Center for Drug Evaluation and Research, U.S. Food and Drug Administration, Silver Spring, MD, United States

**Keywords:** insulin analogs, glucose, pharmacometrics, diabetes, metabolism

## Abstract

Insulin has played an important role in the treatment of diabetes since its discovery in the early 1920s. Initially derived from animal sources, insulin production underwent significant changes with the advent of recombinant DNA technology, allowing for the development of insulin analogs and biosimilar products. Through scientific and technological advances, various types of insulin have been engineered to cater to diverse patient populations, improving the quality, safety, efficacy, and accessibility of these products. There are currently over 50 insulin products approved by the U.S. Food and Drug Administration (FDA), each tailored to meet specific therapeutic needs. This review traces the journey of insulin, from its discovery and purification to recombinant DNA technology, biosimilar developments, and recent advancements in formulation including ultra-rapid formulations and combination therapies. The review also examines the impact of structural and formulation modifications on the pharmacokinetics and pharmacodynamics of insulin, resulting in a range of insulin products with different time-action profiles (rapid-, short-, intermediate-, and long-acting insulins). These technological and manufacturing developments have improved the quality of insulin products available to the public and have made insulin therapy safer, more effective, and more adaptable to individual patient needs, greatly enhancing the management of diabetes over time and patient quality of life.

## 1 Discovery of insulin and animal-derived of insulin products

According to the Centers for Disease Control and Prevention (CDC), diabetes affected over 38.4 million individuals in the US in 2021, representing 11.6% of the population (1). Type 1 diabetes (T1D) is an autoimmune disease characterized by the destruction of pancreatic β-cells, resulting in an inability to produce insulin. For individuals diagnosed with T1D, insulin replacement therapy remains the only viable treatment (2). Type 2 diabetes (T2D), on the other hand, is a complex metabolic disorder associated with insulin resistance and impaired glucose regulation, often linked to obesity and aging. While T2D patients still produce insulin, many require insulin therapy to achieve desired glycemic control, due in part to the development of insulin resistance (3, 4).

In healthy individuals, insulin secretion from the pancreas is precisely regulated by blood glucose levels through a finely tuned feedback mechanism. The pancreatic β-cells within the islets of Langerhans act as glucose sensors, detecting fluctuations in circulating glucose concentrations and responding accordingly (5). After a meal, glucose levels rise, prompting β-cells to secrete insulin in a biphasic manner. The first phase is a rapid release of pre-stored insulin granules that occur within minutes, followed by a more prolonged second phase with sustained insulin secretion and *de novo* insulin synthesis (6). Conversely, during fasting or periods of low glucose availability, insulin secretion decreases to a basal rate sufficient for maintaining glucose homeostasis (7).

Endogenous insulin is stored in pancreatic β-cells as hexamers (insulin clusters of six molecules) stabilized by zinc ions within secretory granules (8). These hexamers serve as an inactive storage form, preventing premature insulin degradation (premature breakdown of insulin molecules that may affects its biological activity) and facilitating controlled release. Upon stimulation by elevated blood glucose, insulin-containing granules undergo exocytosis, releasing insulin into the bloodstream. Once in circulation, the stabilized hexamers dissociate into monomers (individual insulin molecules), converting insulin into its biologically active form (9). Monomeric insulin first exerts its effects on the liver, the primary site of insulin metabolism (10). Hepatic insulin action is crucial for maintaining glucose balance, as insulin suppresses glycogenolysis (breakdown of stored glycogen into glucose) and inhibits gluconeogenesis (production of new glucose); by reducing hepatic glucose output, insulin helps prevent excessive postprandial hyperglycemia (10). The liver also plays a key role in insulin clearance, degrading up to 80% of the insulin it receives before the hormone reaches the systemic circulation (11). The remaining insulin is distributed to peripheral tissues, where insulin facilitates glucose uptake, suppresses triglyceride breakdown in adipose tissue, enhances energy metabolism, and is ultimately cleared by the kidneys (10).

Structurally, insulin is a polypeptide hormone composed of 51 amino acids, arranged in two distinct chains: the A chain (21 amino acids) and the B chain (30 amino acids) (12). These chains are connected by two interchain disulfide bonds (CysA7–CysB7 and CysA20–CysB19), which are necessary for insulin’s stability and bioactivity. The two interchain disulfide bridges, an additional intrachain disulfide bond (CysA6–CysA11) within the A chain along with the A and B chain primary sequences maintain the higher order structure of insulin that ensures binding to specific receptors and concomitant insulin functions (13). Overall, the three-dimensional structure of the polypeptide is essential for insulin’s ability to regulate glucose metabolism effectively.

Since the discovery of insulin in the 1920s, our understanding of diabetes and insulin therapy has advanced significantly (4). Early commercial insulin was extracted from porcine or bovine pancreatic tissue, requiring extensive purification to remove contaminants that could trigger immune responses. Bovine insulin differs from human insulin by three amino acids, while porcine insulin differs by a single amino acid at position B30 (alanine instead of threonine), making the porcine sequence less immunogenic but still capable of eliciting antibody formation and allergic reactions [**Table 1**; (15, 16)]. Early purification methods, such as ethanol precipitation and acid-alcohol extraction, were crude, often leaving behind trace amounts of proinsulin, glucagon, and other pancreatic proteins, contributing to increased immunogenicity (17), and batch-to-batch content variability. By the mid-20th century, insulin purification techniques were optimized with the introduction of gel filtration, ion-exchange chromatography, and crystallization methods, which helped improve the purity of insulin preparations (18). These purification improvements resulted in a 60% reduction in injection site reactions and a 40% decrease in insulin antibody formation compared to earlier preparations, significantly improving patient tolerability and therapeutic outcomes (15, 16). In parallel, formulation science introduced hexamer-stabilizing agents (e.g., zinc, phenol, and protamine) to control insulin release kinetics (the rate at which insulin becomes available in the body). The full sequence of bovine insulin was published in several articles by Frederic Sanger between 1945 and 1955, identifying the peptide amino acid composition (19–26). In 1969, Dorothy Crowfoot-Hodgkin resolved a three-dimensional structure of porcine insulin using X-ray crystallography (27). These breakthroughs along with technological advances paved the way for the development of the first fully synthetic insulin in 1975 by Ciba-Geigy and the production of the first recombinant human insulin using E. coli in 1978 by David Goeddel and colleagues (4). The introduction of recombinant DNA technology in the 1980s revolutionized insulin manufacturing, providing a safer and more consistent alternative to animal-derived insulin while reducing cross-species immunogenicity concerns (4).

**Table 1:** Comparative immunogenicity profile of insulin sources.

Insulin Source	Amino Acid Differences from Human Insulin	Immunogenicity Risk Level	Antibody Formation Rate	References
Bovine	3 amino acids:• A8: Ala → Thr• A10: Ile → Val• B30: Thr → Ala	High	25-30% of patients developed insulin antibodies	*Diabetes Care. 1993;16 Suppl 3:155-65.*Cochrane Database Syst Rev. 2005;2005(1):CD003816.*Diabetologia. 1983;25(6):465-9.
Porcine	1 amino acid:• B30: Ala → Thr	Moderate	10-15% of patients developed insulin antibodies	*Diabetes Care. 1993;16 Suppl 3:155-65.*Diabetologia. 1983;25(6):465-9.
Human (Recombinant)	None (identical sequence)	Minimal	<5% of patients develop insulin antibodies*	*Sci Rep. 2020;10(1):20519.* Diabetes Obes Metab. 2014;16(8):787-95

This table lists the immunogenic potential of animal-derived (bovine and porcine) versus recombinant human insulin. It includes amino acid sequence differences, immunogenicity risks and antibody formation rates. *When insulin antibodies develop with recombinant human insulin, they are typically low-titer and clinically insignificant (14).

## 2 Recombinant DNA technology era and current US FDA-approved insulin products

**Figure 1:**
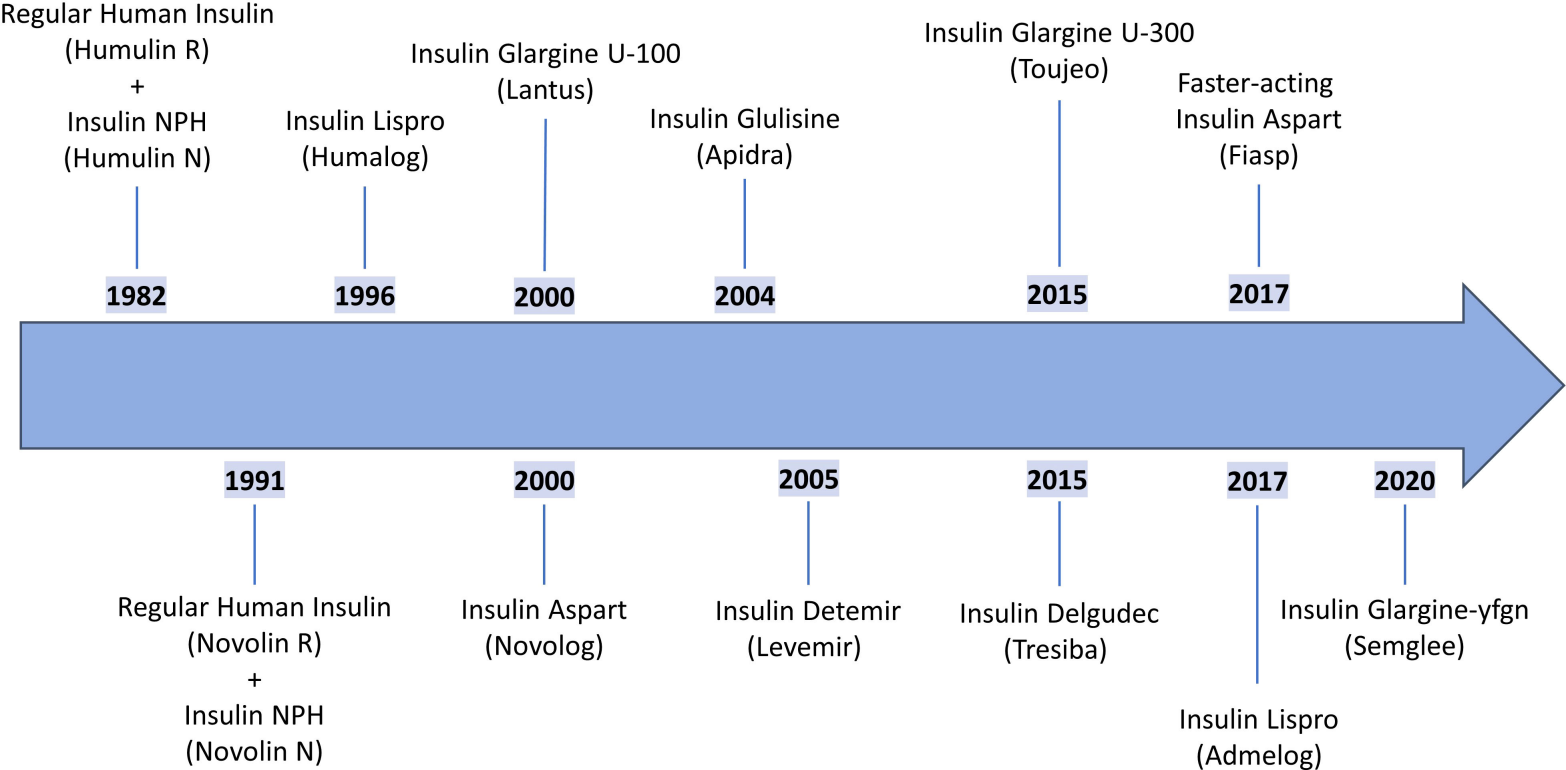
Timeline of US-FDA approval of prevalent insulin products. This chronological timeline illustrates the major milestones in insulin development from the first recombinant human insulin (Humulin R^®^, 1982) through the first biosimilar insulin approved in the USA (Semglee^®^, 2020) and includes key moments for insulin therapy such as the introduction of the first rapid-acting insulin (Humalog^®^, 1996), the first long-acting analog (Lantus^®^, 2000), and the first interchangeable biosimilar (Semglee^®^, 2020). The timeline provides historical context for understanding how insulin therapy has advanced over four decades.

The 1980s and 1990s marked a transformative period in insulin therapy with the advent of genetically modified insulin analogs, designed to optimize absorption, metabolism, and clearance. With utilization of recombinant DNA technology, insulin analogs were engineered to enhance pharmacokinetics, resulting in more predictable glucose control and improved patient outcomes. Utilizing recombinant DNA technology, Eli Lilly introduced the first recombinant human insulin, Humulin R^®^, in 1982 and the first recombinant human insulin NPH, Humulin N, also in 1982 [**Figure 1**, (28, 29)]. The introduction of recombinant human insulin eliminated immunogenicity concerns associated with animal-derived products and provided consistent, reliable insulin therapy (30). This was followed by NovoNordisk in 1991 with a recombinant human insulin, Novolin R^®^, and a recombinant human insulin NPH, Novolin N [**Figure 1**, (31, 32)], expanding patient access to human insulin products.

The first rapid-acting insulin, insulin lispro (Humalog^®^) was released in 1996 by Eli Lilly, followed by Novo Nordisk’s rapid-acting insulin aspart (Novolog^®^) in 2000 (33, 34). In that same year, Sanofi revolutionized basal insulin therapy with the introduction of insulin glargine (Lantus), the first long-acting insulin analog.

**Figure 2:**
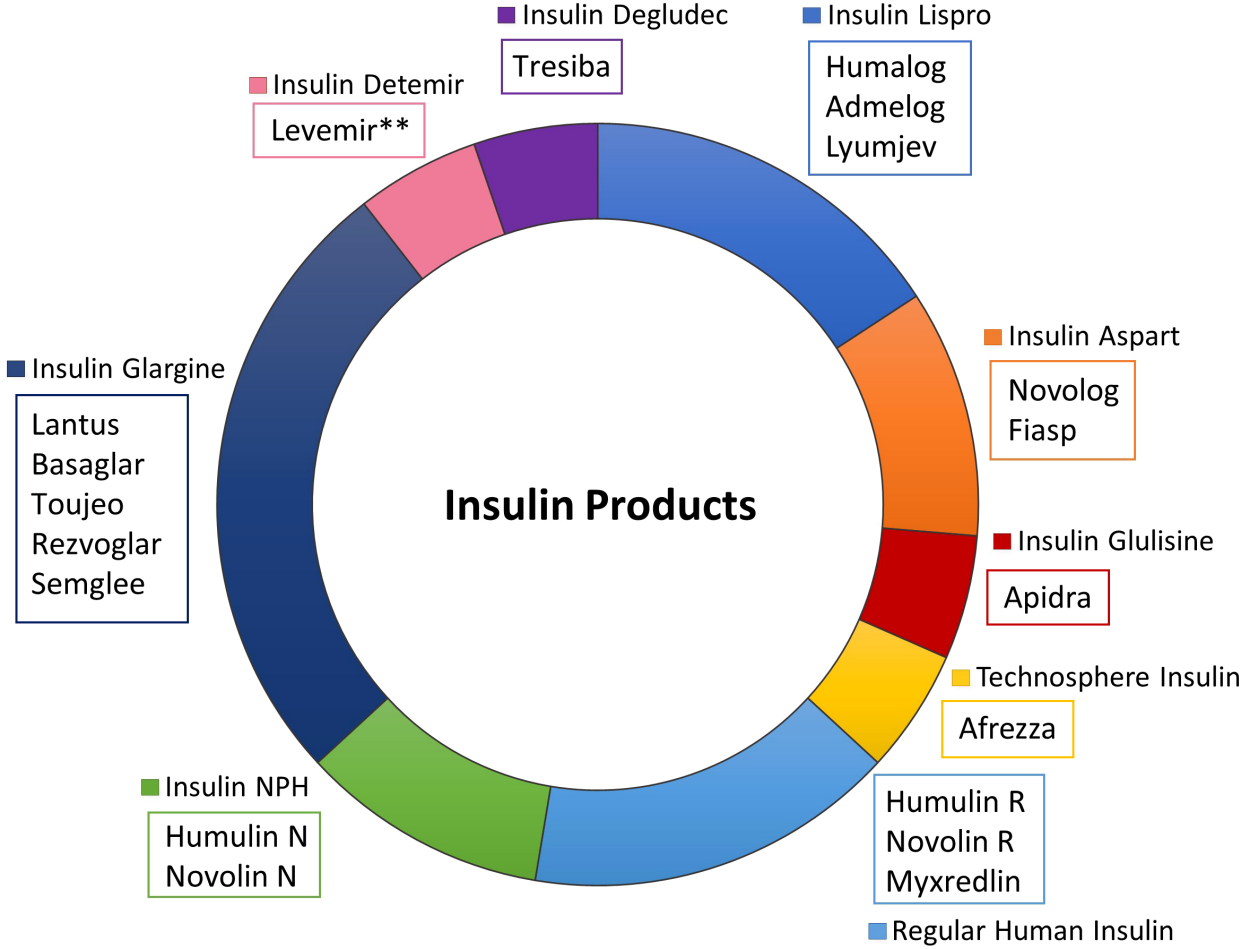
Diagram of prevalent US FDA-approved insulin products. This categorical diagram organizes all major insulin products by their pharmacokinetic classification (rapid-acting, short-acting, intermediate-acting, long-acting, and combination products). Brand names products are grouped by insulin types to illustrate the therapeutic options available within each category, including biosimilar alternatives and discontinued products.

In subsequent years, additional analogs emerged, including insulin glulisine (Apidra^®^), another rapid-acting insulin, as well as long-acting formulations such as insulin detemir (Levemir^®^) and insulin degludec (Tresiba^®^) (**Figure 1**). Multiple variants of these insulins have also been marketed (**Figures 1**, **2**), including several “follow-on” products such as Admelog and Basalgar, which are insulin products that have been approved as biosimilars outside of the USA (35, 36). The first interchangeable biosimilar for insulin glargine, insulin glargine-yfgn U100 (Semglee^®^) from Biocon was approved in 2020, providing alternatives while maintaining therapeutic equivalence (37). A non-exclusive timeline of prevalent US-FDA approved insulin products is shown in **Figure 1** and highlights key milestones in the evolution of insulin therapy. A diagram presenting a categorized overview of prevalent commercial insulin products, grouped by insulin type is shown in **Figure 2**.

## 3 Effect of structural and formulation changes on the function of insulin products

Like many proteins, insulin in aqueous solutions can degrade through deamidation (loss of amino group) or aggregation (when insulin molecules clump together to form larger structures). At low pH, deamination has been shown to primarily affect asparagine at A21, whereas under neutral or alkaline conditions, asparagine at B3 has been observed to be more susceptible to deamination (38). To maintain stability and potency, insulin production requires purification steps to eliminate impurities and tightly controlled formulations to maintain hormone stability and potency. Overall, advances in purification methodologies, including chromatography and filtration techniques, have been instrumental in producing more stable and potent insulin products with improved shelf-life and reduced immunogenic potential (4). Modern commercial insulin and insulin analog formulations typically include stabilizing agents such as zinc, preservatives, and buffers (**Table 2**). Upon subcutaneous injection, these additives facilitate the controlled dissociation of hexameric insulin (storage form) into its monomeric state (active form), enhancing insulin’s absorption into the bloodstream and uptake by the liver (39).

**Table 2:** Formulations of prevalent US FDA-approved insulin products.

Types of Insulin				
Insulin, Active Ingredient	Structural Change from Human Insulin	Formulation, Inactive Ingredients	Commercial Name	Manufacturer
Rapid-Acting Insulin				
Insulin Lispro	Inversion of proline at B28 and lysine at B29	Glycerin, meta-cresol, zinc oxide, disodium phosphate, **phenol**, water for injection, hydrochloric acid and/or sodium hydroxide	**Humalog®**	Eli Lilly
			**Admelog®**	Sanofi
Insulin Lispro-aabc		Glycerin, meta-cresol, zinc oxide, disodium phosphate, **phenol**, water for injection, **treprostinil, citrate,** hydrochloric acid and/or sodium hydroxide	**Lyumjev®**	Eli Lilly
Insulin Aspart	Replacement of proline at B28 with aspartic acid	Glycerin, meta-cresol, sodium chloride, disodium phosphate, **phenol**, water for injection, hydrochloric acid and/or sodium hydroxide	**Novolog®**	Novo Nordisk
		Glycerin, meta-cresol, sodium chloride, disodium phosphate, **phenol**, **niacinamide, L-arginine,** water for injection, hydrochloric acid and/or sodium hydroxide	**Fiasp®**	Novo Nordisk
Insulin Aspart-szjj		Meta-cresol, sodium chloride, zinc chloride, **phenol**, **polysorbate 20**, water for injection, hydrochloric acid and/or sodium hydroxide	**Merilog®**	Sanofi
Insulin Glulisine	• Replacement of asparagine at B3 with lysine• Replacement of lysine at B29 with glutamic acid	Meta-cresol, sodium chloride, water for injection, **tromethamine**, **polysorbate 20,** hydrochloric acid and/or sodium hydroxide	**Apidra®**	Sanofi
Technosphere Insulin(Regular Human Insulin)	No changes from human insulin	**Fumaryl diketopiperazine, polysorbate 80**	**Afrezza®**	MannKind Corporation
Short-Acting Insulin				
Regular Human Insulin	No changes from human insulin	Glycerin, meta-cresol, zinc oxide, water for injection, hydrochloric acid and/or sodium hydroxide	**Humulin R®**	Eli Lilly
		Glycerin, meta-cresol, zinc oxide, water for injection, hydrochloric acid and/or sodium hydroxide	**Novolin R®**	Novo Nordisk
		Disodium phosphate, **sodium phosphate monobasic monohydrate**, **sodium chloride**, water for injection	**Myxredlin®**	Baxter Healthcare
Intermediate-Acting Insulin				
Insulin Neutral Protamine Hagedorn (NPH) (Isophane)	No changes from human insulin	**Protamine sulfate**, glycerin, meta-cresol, zinc oxide, disodium phosphate, **phenol**, water for injection, hydrochloric acid and/or sodium hydroxide	**Humulin N®**	Eli Lilly
		**Protamine sulfate**, glycerin, meta-cresol, zinc, disodium phosphate, **phenol**, water for injection, hydrochloric acid and/or sodium hydroxide	**Novolin N®**	Novo Nordisk
Long-Acting Insulin				
Insulin Glargine	• Replacement of asparagine at A21 with glycine • Addition of two arginines to C-terminus of the B-chain	Glycerol, meta-cresol, zinc, **polysorbate 20**, water for injection, hydrochloric acid and/or sodium hydroxide	**Lantus®**	Sanofi
		Glycerin, meta-cresol, zinc oxide, water for injection, hydrochloric acid and/or sodium hydroxide	**Basaglar®**	Novo Nordisk
		Glycerin, meta-cresol, zinc, water for injection, hydrochloric acid and/or sodium hydroxide	**Toujeo®**	Sanofi
Insulin Glargine-aglr		Glycerin, meta-cresol, zinc oxide, water for injection, hydrochloric acid and/or sodium hydroxide	**Rezvoglar®**	Eli Lilly
Insulin Glargine-yfgn		Glycerol, meta-cresol, zinc chloride, **polysorbate 20,** water for injection, hydrochloric acid and/or sodium hydroxide	**Semglee®**	Biocon Biologics
Insulin Detemir	• Deletion of threonine at B30 • Addition of 14-carbon fatty acid chain at lysine at B29	Glycerin, meta-cresol, zinc, disodium phosphate, **sodium chloride**, **phenol**, water for injection, hydrochloric acid and/or sodium hydroxide	Levemir®**	Novo Nordisk
Insulin Degludec	• Deletion of threonine at B30 • Addition of 16-carbon fatty acid chain at lysine at B29	Glycerin, meta-cresol, zinc, **phenol**, water for injection, hydrochloric acid and/or sodium hydroxide	**Tresiba®**	Novo Nordisk

This table details the specific amino acid modifications, formulation components, and mechanisms of action for major insulin products approved in the United States. The table is organized by insulin category (rapid-acting, short-acting, intermediate-acting, long-acting, and combination products). The bolded inactive ingredients refer to the differences in formulation when compared to those of the originator insulin products.

Commercial insulin products differ in structure, formulation and/or presentation (**Table 2**). These differences can be observed by analytical procedures sensitive to alteration in higher order structure (40–43). In addition, these differences can be observed using pharmacometrics data, such as pharmacokinetics (PK) and pharmacodynamics (PD), which are used to define the time-action profile of insulin products [ (44), **Tables 3**–**5**]: i) onset of action (the time it takes for the blood glucose to lower after injection); ii) time to peak (the time taken to reach maximum effect); and iii) duration of action (how long the effects last after an injection) (45). The ability of insulin to lower blood glucose depends on its ability to transition from hexameric storage forms to active monomers. Since only insulin monomers can pass through the capillary walls, the time to onset depends on the strength of the interactions that bind the insulin hexamers in the subcutaneous environment. When these interactions are strong, the time to onset and the duration of action can take longer as the hexamers need to be broken down into monomers before entering the bloodstream (45). Conversely, weaker interactions facilitate faster monomer release, resulting in a more rapid onset and shorter duration of action [refer to these comprehensive reviews for detailed visual comparisons of onset, peak, and duration among different insulin types (44, 45)]. To optimize insulin PK/PD properties, structural modifications and formulation advancements have been introduced. For example, rapid-acting insulins incorporate amino acid substitutions that reduce self-association, accelerating monomer release, while long-acting insulins are engineered with fatty acid modifications or amino acid substitutions that enhance hexamer stability and prolong absorption [refer to **Table 2** and (12)]. Additionally, formulation excipients such as zinc, phenol, and protamine are used to modulate insulin aggregation and release kinetics [refer to **Table 2** and (46)]. Based on their time-action profiles, insulin products can be categorized into four major groups: rapid-acting, short-acting, intermediate-acting, and long-acting insulins (44). These classifications guide clinical decision-making, allowing for the selection of insulin therapies that align with patient-specific glucose management needs.

**Table 3:** Time action profile of US FDA-approved rapid-acting insulin products.

Types of Insulin						
Type of Insulin / Structural Change	Commercial Name	Dosage Form	Route of Administration	Onset	Peak	Duration
Rapid-Acting Insulin						
Insulin Lispro B28: Pro → Lys B29: Lys→Pro	**Humalog®**	Injectable	s.c. Injection (U-100, U-200)	22 min	1-3 hrs	6 hrs
		Infusion	s.c. (Pump, U-100) i.v. (Diluted U-100)*			
							**Admelog®**	Injectable	s.c. Injection (U-100)	20 min	1-3 hrs	6 hrs
	Infusion	s.c. (Pump, U-100) i.v. (Diluted U-100)*										
			Insulin Lispro-aabc B28: Pro → Lys B29: Lys→Pro	**Lyumjev®**	Injectable	s.c. Injection (U-100, U-200)		17 min	1-2 hrs				4 hrs
	Infusion	s.c. (Pump, U-100) i.v. (Diluted U-100)*											
Insulin Aspart B28: Pro → Asp					**Novolog®**	Injectable	s.c. Injection (U-100)			15 min	1-3 hrs	3-5 hrs	
	Infusion	s.c. (Pump, U-100) i.v. (Diluted U-100)*											
			**Fiasp®**	Injectable		s.c. Injection	15-20 min	1.5-2.5 hrs	5-7 hrs			
	Infusion	s.c. (Pump, U-100) i.v. (Diluted U-100)*							
				Insulin Aspart-szjj B28: Pro → Asp	**Merilog®**	Injectable				s.c. Injection (U-100)	15 min	1-3 hrs	3-5 hrs
	Insulin Glulisine B3: Asn → Lys B29: Lys → Glu	**Apidra®**	Injectable				s.c. Injection (U-100)	15-30 min	0.5-1 hr					4 hrs
Infusion				s.c. Injection (Pump, U-100) i.v. (Diluted U-100)*										
			Technosphere Insulin		**Afrezza®**	Powder, Metered	Oral Inhalation (4, 8 or 12 units)			< 5 min	15 min	3 hrs		

This table provides the pharmacokinetic parameters (onset, peak, and duration), route of administration, dosage form and commercial names for FDA-approved rapid-acting insulin products. It also includes the structural differences of the analogs compared to human insulin.

s.c.; subcutaneous; i.v.; intravenous, hr(s): hour(s); min: minutes; *intravenous infusion.

**Table 4:** Time action profile of US FDA-approved short-, intermediate-, and long-acting insulin products.

Types of Insulin						
Type of Insulin / Structural Change	Commercial Name	Dosage Form	Route of Administration	Onset	Peak	Duration
Short-Acting Insulin						
Regular Human Insulin	**Humulin R®**	Injectable	s.c. Injection (U-100, U-500)	~ 1 hr	2-4 hrs	5-8 hrs
		Infusion	i.v. (Diluted U-100)*			
	**Novolin R®**	Injectable	s.c. Injection (U-100)	~ 1 hr	2-4 hrs	5-8 hrs
		Infusion	i.v. (Diluted U-100)*			
	**Myxredlin®**	Solution	i.v. (U-100)*	~ 1 hr	2-4 hrs	5-8 hrs
Intermediate-Acting Insulin						
Insulin Neutral Protamine Hagedorn (NPH) (Isophane)	**Humulin N®**	Injectable	s.c. Injection (U-100)	1-2 hrs	4-10 hrs	14-24 hrs
	**Novolin N®**	Injectable	s.c. Injection (U-100)	1-2 hrs	4-10 hrs	14-24 hrs
Long-Acting Insulin						
Insulin Glargine A21: Asn → Gly Addition of two Arg to C-terminus of the B-chain	**Lantus®**	Injectable	s.c. Injection (U-100)	3-4 hrs	No peak	24 hrs
	**Basaglar®**	Injectable	s.c. Injection (U-100)	3-4 hrs	No peak	24 hrs
	**Toujeo®**	Injectable	s.c. Injection (U-300)	6 hrs	No peak	36 hrs
Insulin Glargine-aglr A21: Asn → Gly Addition of two Arg to C-terminus of the B-chain	**Rezvoglar®**	Injectable	s.c. Injection (U-100)	3-4 hrs	No peak	24 hrs
Insulin Glargine-yfgn A21: Asn → Gly Addition of two Arg to C-terminus of the B-chain	**Semglee®**	Injectable	s.c. Injection (U-100)	3-4 hrs	No peak	24 hrs
Insulin Detemir B30: Deletion of Thr B29: Addition of 14-carbon fatty acid chain at Lys	Levemir®**	Injectable	s.c. Injection (U-100)	1-2 hrs	6-8 hrs	20-24 hrs
Insulin Degludec B30: Deletion of Thr B29: Addition of 14-carbon fatty acid chain at Lys	**Tresiba®**	Injectable	s.c. Injection (U-100, U-200)	1-2 hrs	No peak	42 hrs

This table provides the pharmacokinetic parameters (onset, peak, and duration), route of administration and commercial names for FDA-approved short, intermediate and long-acting insulin products. It also includes the structural differences of the analogs compared to human insulin.

s.c.; subcutaneous; i.v.; intravenous, hr(s): hour(s); min: minutes;*intravenous infusion; **discontinued.

**Table 5:** Time action profile of US FDA-approved combination insulin products.

Types of Insulin: Combination						
Commercial Name	Type of Insulin	Dosage Form	Route of Administration	Onset	Peak	Duration
Intermediate- and Rapid-Acting						
Humalog Mix 75/25	75% Insulin Lispro Protamine 25% Insulin Lispro	Injectable	s.c. Injection (U-100)	15-30 min	0.5-2.5 hrs	14-24 hrs
Humalog Mix 50/50	50% Insulin Lispro Protamine 50% Insulin Lispro	Injectable	s.c. Injection (U-100)	15-30 min	0.5-3 hrs	14-24 hrs
NovoLog Mix 70/30	70% Insulin Aspart Protamine 30% Insulin Aspart	Injectable	s.c. Injection (U-100)	6-12 min	1-4 hrs	18-24 hrs
Intermediate- and Short-Acting						
Humulin 70/30	70% NPH 30% Human Insulin	Injectable	s.c. Injection (U-100)	30-60 min	2-12 hrs	10-16 hrs
Novolin® 70/30	70% NPH 30% Human Insulin	Injectable	s.c. Injection (U-100)	30-60 min	2-12 hrs	10-16 hrs
Long – and Rapid-Acting						
Ryzodeg 70/30*	70% Insulin Degludec 30% Insulin Aspart	Injectable	s.c. Injection (U-100)	15 min	2.3 hrs	> 24 hrs
Long-Acting Insulin and Glucagon-like peptide-1 (GLP-1) Agonists						
Soliqua 100/33	100U Insulin Glargine 33 mcg/mL Lixisenatide	Injectable	s.c. Injection	1-3 hrs	2.5-3 hrs	20-24 hrs
Xultophy 100/3.6	100U Insulin Degludec 3.6 mg/mL Liraglutide	Injectable	s.c. Injection	1-2 hrs	8-11 hrs	> 24 hrs

This table covers all combination insulin products, including traditional premixed formulations, ultra-long-acting combinations, and innovative fixed-ratio insulin/GLP-1 receptor agonist combinations.

s.c.; subcutaneous; hr(s): hour(s); min: minutes; *discontinued

### 3.1 Short- and rapid-acting insulin

Endogenous human insulin has an onset of action of approximately 30–60 min, reaches peak activity within 2–4 h, and maintains its glucose-lowering effect for 5–8 h (44). The first recombinant human insulin products (Humulin R^®^ and Novolin R^®^) exhibited a slower increase in serum insulin concentration and a more prolonged duration of action compared to endogenous insulin, limiting the capability of Humulin R^®^ and Novolin R^®^ to effectively manage postprandial glucose spikes (28, 31, 44) (**Figure 1** and **Table 3**). Indeed, both Humulin R^®^ and Novolin R^®^ required injection 30 minutes prior to food intake, which made it difficult for patients to adequately time delivery prior to meals. Efforts were then made to develop insulins with a faster onset of action. Rapid-acting insulin analogs, including insulin lispro, insulin aspart, insulin glulisine, and insulin technosphere, were engineered through amino acid modifications in the B chain of the insulin molecule [**Tables 2**, **3** and (44)]. These modifications disrupted insulin dimer and hexamer formation, facilitating the rapid dissociation of insulin monomers and enhancing absorption of insulin into the bloodstream (47). For instance, the inversion of the proline-lysine amino acid sequence at positions B28 and B29 produced insulin lispro, while replacing the proline at B28 with aspartic acid led to insulin aspart. Additionally, substituting the asparagine at B3 with lysine and the lysine at B29 with glutamic acid resulted in insulin glulisine (**Table 1**). These variations reduced the strength of hydrogen bonds that contribute to the stability of insulin dimers, leading to faster dissociation of insulin hexamers and enhanced absorption of monomeric insulin (48). Insulin lispro is commercially available under the brand names Humalog^®^ and Admelog^®^, with Admelog^®^ approved as a follow-on of the former. Insulin lispro analogs improved mealtime glucose management by reducing postprandial glucose excursions by 25% and allowing patients greater flexibility in meal timing (49). In 2020, insulin lispro-aabc was approved as Lyumjev^®^ and incorporates treprostinil and citrate to accelerate absorption and reduce time to peak action, providing patients with the fastest-acting insulin available at the time (50) (**Tables 2**, **3**).

Insulin aspart was developed by substituting proline at B28 with aspartic acid. Insulin aspart is commercially available as Novolog^®^, Fiasp^®^, and Merilog ^®^. Fiasp^®^, which was approved by the FDA in 2017, contains L-arginine and niacinamide to enhance stability and absorption, offering improved postprandial glucose control compared to standard rapid-acting formulations [ (51);**Table 2**]. Merilog^®^ (insulin-aspart-szjj), an insulin aspart analog and biosimilar of Novolog^®^, was approved in 2025 as the first rapid-acting biosimilar product in the USA, offering an alternative for patients requiring rapid-acting insulin therapy (52).

Insulin glulisine was approved as Apidra^®^ in 2004 (53). Unlike insulin lispro or aspart, insulin glulisine does not contain zinc, which reduces hexamer formation and accelerates absorption (**Table 2**). Instead, the addition of polysorbate 20 is known to increase the solubility and prevent aggregation of the insulin glulisine, while tromethamine helps stabilize the pH of the formulation (54) (**Table 2**).

In 2014, an ultra-rapid-acting, inhalable insulin formulation known as technosphere insulin (Afrezza^®^) was introduced [**Table 2**, (55)]. This dry powder insulin is aerosolized through an inhaler and absorbed via the lungs, allowing for a much faster onset of action compared to conventional rapid-acting insulins. However, the technosphere insulin absorption mechanism also results in a shorter duration of action, necessitating more frequent dosing to maintain postprandial glucose control (45, 51). Technosphere insulin is also contraindicated in patients with chronic lung disease, asthma, or COPD due to potential pulmonary complications, and requires pulmonary function monitoring, limiting its clinical applicability (55).

Due to their rapid onset of action, short-acting and rapid-acting insulins—collectively referred to as prandial insulins—are designed to be administered at mealtime to effectively manage postprandial glucose excursions. The development of these analogs has significantly improved glycemic control by providing more physiologically relevant insulin delivery and more closely mimicking endogenous insulin secretion patterns. These rapid-acting insulins (with the exception of Afrezza^®^) are also most often used for insulin pump therapy (refer also to **Table 3**), which decreases the need for multiple injections per day and helps improve the glycemic control of patients.

### 3.2 Intermediate acting insulins

Basal insulin is essential in maintaining stable blood glucose levels in between meals, or during periods of fasting. The first commercially available insulins were derived from bovine or porcine sources and were limited to short-acting formulations. Consequently, patients required multiple daily injections to maintain their blood glucose at adequate levels (2). To address these limitations, formulation modifications were investigated to extend the insulin’s duration of action. In 1936, it was discovered that adding protamine, a fish-derived protein, resulted in crystallization of insulin hexamers, thereby slowing monomer dissociation, and delaying absorption into the bloodstream (48, 56). Aside from protamine, other additives such as globin protein and zinc, surfen, and lente with zinc, incorporated into insulin formulations also slowed monomer dissociation and delayed absorption of insulin (44, 57). These findings led to the development of the first intermediate-acting insulin: Neutral Protamine Hagedorn (NPH) insulin, which was formulated with protamine. Although NPH was initially introduced using animal-sourced insulin, the drug is now manufactured using recombinant human insulin and is commercially available as Humulin N^®^ and Novolin N^®^ [**Table 2** and (29, 32)]. Due to its formulation, NPH insulin exhibits a prolonged duration of action, typically lasting 14–24 h, allowing patients to maintain glycemic control overnight (47, 58) (**Table 4**). However, a single NPH injection is not sufficient to mimic daily physiological basal insulin levels, and hence patients require twice-daily administration to reach adequate basal threshold. Current usage patterns show NPH insulin is primarily used in resource-limited settings due to its lower cost, though its variably absorption and peak effect at 4–6 hours can increase hypoglycemia risk compared to more recent analogs (44). The need to have multiple injections per day and the unpredictable peak led to the development of long-acting insulins, which have since largely replaced intermediate-acting insulin products.

### 3.3 Long-acting insulins

The development of long-acting insulin analogs provided a stable basal insulin supply, reducing the need for frequent injections and improving the stability in glucose control with reduced day-to-day variability (53, 59, 60). These analogs are designed to mimic endogenous basal insulin secretion by exhibiting prolonged pharmacokinetics (PK) and minimal peak activity.

The first long-acting insulin analog, insulin glargine, was introduced in 2000. Insulin glargine incorporates two key structural modifications: substitution of asparagine with glycine at position A21 and the addition of two arginine residues at B30 [**Table 2** and (61)]. The glycine substitution enhances the stability of insulin glargine in acidic formulations, while the arginine residues shift the isoelectric point, making the insulin less soluble at physiological pH (44). Upon subcutaneous (s.c.) injection, insulin glargine precipitates in the neutral pH environment of the s.c. tissue, allowing a slow and continuous release of insulin monomers into circulation (62). This mechanism provides a relatively flat PK profile with minimal peak activity and maintains glucose-lowering effects for up to 24 h and more closely resembling endogenous basal insulin secretion, reducing nocturnal hypoglycemia by 40% compared to NPH insulin, while improving patient compliance through once-daily dosing (63)(**Table 4**). Insulin glargine is commercially available under the brand names Lantus^®^, Basaglar^®^, Rezvoglar^®^, Toujeo^®^, and Semglee^®^, the first US biosimilar of Lantus^®^ [**Table 4** and (36, 37, 64, 65)]. Toujeo^®^ is a higher-concentration formulation (U-300) of insulin glargine than the other marketed analogs and provides a longer duration of action (up to 36 h) with even flatter glucose-lowering profile (65). Insulin detemir is another type of long-acting insulin (59). Approved in 2005, Insulin detemir utilizes a different mechanism to prolong its duration of action. Insulin detemir is structurally modified by the deletion of threonine at B30 and the attachment of a 14-carbon fatty acid (myristic acid) to lysine at B29 [**Tables 2**, **4**; (66)]. This acylation enhances self-association, promoting dihexamer formation, and enables reversible binding to serum albumin, which further delays insulin clearance from circulation. As a result, insulin detemir has a gradual onset of action, a peak at approximately 6 h, and a duration of 20–24 h, although some patients may require twice-daily administration for optimal glycemic control (67). Insulin detemir is marketed as Levemir^®^, although this product is to be discontinued starting in 2025. Patients currently using Levemir^®^ should transition to alternative long-acting insulins such as insulin glargine or insulin degludec under medical supervision to maintain glucose control (68).

Introduced in 2015, insulin degludec is an ultra-long-acting insulin analog, designed to provide even more stable basal insulin levels and greater dosing flexibility than other available versions (60, 69, 70). Similar to insulin detemir, insulin degludec features the deletion of threonine at B30, but instead of myristic acid, insulin degludec is conjugated to a 16-carbon fatty acid (hexadecanedioic acid) via a glutamic acid linker at B29 (69). insulin degludec’s formulation contains zinc and phenol, which facilitate the formation of insulin hexamers in solution. Once injected, the depletion of phenol promotes the self-association of these hexamers into linear multi-hexamers, creating a subcutaneous depot (70). The gradual dispersion of zinc ions leads to a controlled release of monomers, ensuring a slow and sustained insulin release. Additionally, the fatty acid modification enables albumin binding, further prolonging insulin availability. Insulin degludec has an onset of action within 1–2 h, lacks a distinct peak, and maintains glucose-lowering effects for up to 42 h, reducing the risk of hypoglycemia by 25% compared to insulin glargine and allowing greater dosing flexibility with timing variations of up to 8 hours (71). Insulin degludec is commercially available as Tresiba^®^.

### 3.4 Combination insulins

For individuals with diabetes requiring both prandial and basal insulin throughout the day, managing multiple injections can be burdensome and increase the risk of dosing errors. Additionally, not all insulin formulations can be mixed due to differences in their chemical properties and mechanisms of action. To address these challenges, pharmaceutical companies developed premixed insulin formulations, which combine rapid- or short-acting insulins with intermediate-acting insulins in fixed ratios. These formulations simplify treatment regimens, improve patient compliance, and provide both immediate postprandial glucose control and sustained basal insulin coverage.

Premixed insulins offer a convenient solution for balancing glucose control across different periods of the day (72). Among the first approved combinations were Humalog^®^ Mix 75/25 and Humalog^®^ Mix 50/50, both introduced in 1999 [**Table 5**; (73, 74)]. These formulations contain insulin lispro protamine suspension, an intermediate-acting insulin, combined with insulin lispro, a rapid-acting insulin. Similarly, Novolog^®^ Mix 70/30 (75) combines insulin aspart protamine suspension with insulin aspart, while Humulin^®^ 70/30 and Novolin^®^ 70/30 utilize NPH insulin as the intermediate component, paired with regular human insulin (**Table 5**). These combinations offer a structured approach to glucose management by providing sustained insulin action between meals and at night while addressing postprandial glucose spikes. While convenient, premixed formulations offer limited dosing flexibility and may increase hypoglycemia risk due to fixed ratios that cannot be adjusted for varying carbohydrate intake or activity levels (72). Patients require consistent meal timing and carbohydrate content for optimal safety and efficacy. More recently, Ryzodeg^®^ 70/30, which pairs ultra-long-acting insulin degludec with rapid-acting insulin aspart, has emerged as an alternative (76). Unlike other premixed insulins, Ryzodeg^®^ provides extended basal coverage for up to 42 h, allowing for greater dosing flexibility and reduced injection frequency.

The introduction of glucagon-like peptide-1 receptor agonists (GLP-1 RAs) further revolutionized diabetes management by stimulating glucose-dependent insulin secretion, suppressing glucagon release, and slowing gastric emptying, thereby improving both postprandial glucose control and weight regulation (77). Given their complementary mechanisms of action, insulin and GLP-1 RAs have been combined into fixed-ratio formulations to simplify treatment and enhance glycemic control while minimizing weight gain and the risk of hypoglycemia. Among these, Soliqua^®^ 100/33 and Xultophy^®^ 100/3.6 have been approved (78, 79). Soliqua^®^ 100/33 pairs insulin glargine (Lantus^®^) with lixisenatide (Adlyxin^®^) to provide sustained basal insulin activity alongside a GLP-1 RA that improves postprandial glucose regulation. Similarly, Xultophy^®^ 100/3.6 combines ultra-long-acting insulin degludec (Tresiba^®^) with liraglutide (Victoza^®^), offering extended glucose control with fewer daily injections and more flexibility in dosing (**Table 4**). Despite their clinical benefits, GLP-1 RA/insulin combinations face accessibility challenges (potentially due to higher costs or variable insurance coverage) limiting their adoption in clinical practice.

## 4 Conclusion

Diabetes affects more than 39 million people in the United States and imposes an estimated annual cost of $412.9 billion (as of 2022), representing a significant economic burden through increased medical expenses, lost productivity, and reduced quality of life (80). Given insulin’s critical role in diabetes management, extensive efforts have been made to improve purification and develop formulations that better align with patients’ physiological needs. Advances in insulin therapy have led to a diverse range of products, from short-acting and rapid-acting insulins designed to mimic endogenous postprandial insulin secretion, to intermediate- and long-acting formulations that provide stable basal insulin levels (**Tables 2**-**4**). Structural modifications, such as amino acid substitutions and acylation, influence self-association and receptor binding, while formulation components like zinc, phenol, and protamine regulate aggregation and release kinetics (**Table 1**). These advancements have not only enhanced glycemic control but have also improved patient compliance by reducing injection frequency and minimizing the risk of hypoglycemia. However, insulin therapy still faces significant limitations. Patient variability in absorption, metabolism, and insulin sensitivity creates challenges in achieving optimal glycemic control for all individuals (62, 81, 82). Adherence challenges persist, particularly with complex regimens requiring multiple daily injections, timing considerations, and glucose monitoring. Additionally, the risk of hypoglycemia, weight gain, injection site reactions, and lack of physiologic insulin delivery patterns continue to impact patient quality of life and treatment satisfaction. The future of insulin therapy promises exciting innovations that address current limitations. Weekly insulin formulations currently in Phase III clinical trials aim to reduce injection frequency to once per week, potentially enhancing patient compliance and quality of life while addressing adherence challenges and reducing injection site complications (83). Smart delivery systems in various development stages from preclinical to early clinical trials, including closed-loop insulin pumps and glucose-responsive insulin formulations, are being developed to provide automated, physiologic insulin delivery that minimizes hypoglycemia risk and reduces the need of additional glucose monitoring (84, 85). Oral insulin formulations currently in Phase II/III clinical trials, despite decades of challenges, continue to show promise with novel delivery technologies and absorption enhancers that could eliminate injection-related complications (86, 87). Additionally, personalized insulin therapy approaches using pharmacogenomics and artificial intelligence may optimize dosing and timing for individual patients, minimizing adverse effects while maximizing therapeutic benefits (88, 89). With numerous recombinant insulin innovators and biosimilar products currently marketed in the USA, ongoing manufacturing and product quality research continue to refine insulin therapies, addressing the clinical and economic burden of diabetes while improving the quality of insulin products and, by extension, the quality of life for millions of patients. The continued evolution of insulin therapy represents one of medicine’s greatest success stories, with future innovations promising even greater improvements in diabetes care and patient outcomes.
